# Recommendations for Improving Surveillance of Congenital Anomalies in Europe Using Healthcare Databases

**DOI:** 10.1111/ppe.13173

**Published:** 2025-01-28

**Authors:** Maria Loane, Joan K. Morris, Ester Garne

**Affiliations:** ^1^ Institute of Nursing and Health Research Ulster University Belfast UK; ^2^ School of Health & Medical Sciences City St George's University of London London UK; ^3^ Department of Paediatrics and Adolescent Medicine Lillebaelt Hospital, University Hospital of Southern Denmark Kolding Denmark

**Keywords:** congenital anomalies, healthcare databases, linkage, quality, recommendations, surveillance

## Abstract

**Background:**

Although accessing administrative data in healthcare databases may be a more time‐efficient and cost‐effective method of conducting surveillance, there is evidence suggesting that administrative data alone are not sufficient for population‐based surveillance of congenital anomalies.

**Objective:**

To propose recommendations to maximise the potential use of healthcare databases for surveillance of congenital anomalies based on our data linkage experiences and results from the EUROlinkCAT study.

**Methods:**

EUROlinkCAT is a population‐based cohort study of 99,416 children with anomalies born between 1995 and 2014. The congenital anomaly case records of children in 11 European congenital anomaly (EUROCAT) registries (eight countries) were linked to standardised administrative healthcare data (birth records, death records and hospital discharge records) to evaluate mortality and morbidity outcomes in these children. Overall, 97% of children with anomalies were successfully matched to their records in their national or regional administrative databases. Recommendations to improve surveillance of anomalies when using healthcare data were developed through establishing and analysing data from this cohort.

**Results:**

The primary recommendation is to develop systems to report anomalies diagnosed in foetuses who undergo a termination and link these data to their mothers. Each liveborn baby must be assigned a permanent unique identification number at birth to enable accurate linkage across healthcare databases. Implementing and improving existing algorithms to discriminate between major anomalies and suspected or minor anomalies will improve accuracy in coding. Heterogeneity in coding anomalies will improve by avoiding the use of ‘unspecified’ or ‘other specified’ codes in hospital databases. Relaxation of country‐specific regulations concerning the suppression of small numbers are necessary to enable data to be combined across European countries.

**Conclusion:**

Implementation of these recommendations will enable the information in electronic healthcare databases, in conjunction with Congenital Anomaly registries, to be fully exploited and hence will improve the surveillance of anomalies in children.

## 1 Background

Epidemiological surveillance of congenital anomalies is an important public health activity as anomalies represent a major cause of mortality and morbidity in infants and children. Surveillance aims to identify increases in the prevalence of anomalies that may be due to maternal exposures to teratogenic medications or other environmental teratogens during the first trimester of pregnancy. Many countries conduct surveillance of anomalies by establishing population‐based registries actively collecting data on cases of congenital anomaly using multiple ascertainment sources. Accessing routinely collected data in healthcare databases may be a more time‐efficient and therefore cost‐effective method of conducting such surveillance. However, healthcare databases vary in their aim, function and quality of coding systems, which influences the validity of the recorded data. Research evidence indicates that administrative healthcare data are not currently sufficient for surveillance of anomalies [1, 2].

Until recently, there has been limited population‐based information on the survival, hospital stays, surgical procedures and use of medications in children with anomalies in Europe. The EUROlinkCAT project linked data on children with major anomalies recorded in European congenital anomaly (EUROCAT) registries to administrative data in regional or national health care databases to bridge this research gap [3]. Christen and Schnell [4] noted that there are few published papers describing the experiences and challenges of using administrative data for research and that such papers would hugely benefit others conducting data linkage studies. The aim of this study was to propose recommendations to maximise the potential use of healthcare databases for surveillance of anomalies based on our data linkage experience and results from the EUROlinkCAT study.

## 2 Methods

EUROlinkCAT was a large, linked population‐based cohort study of 99,416 children with major congenital anomalies born between 1995 and 2014. Seventeen European population‐based EUROCAT registries linked their standardised data on children with anomalies to their national/regional mortality data, 11 to hospital discharge databases and seven to prescription data to evaluate mortality and morbidity outcomes in these children (Table 1) [5, 6]. Of the registries that linked to hospital discharge databases and prescription data, seven also included data on children without anomalies born during the same time period and from the same population area covered by the registry. EUROCAT registries use multiple sources of ascertainment to identify and verify congenital anomaly cases, and data are standardised according to EUROCAT guidelines [7].

**TABLE 1:** Source of healthcare data available for linkage in European regions/countries in the EUROlinkCAT study.

EUROCAT registry	Single data access provider for all healthcare data sources used in study	Source of mortality data	Source of morbidity data	Source of prescription data
Belgium: Antwerp	No	Flemish Agency for Care and Health, Belgian Mortality Registry		
Croatia: Zagreb	No		Republic of Croatia Bureau of Statistics or Croatian Health Insurance System	
Denmark: Funen	Statistics Denmark	Registry for cause of deaths	The Danish National Patient Registry (DNPR)	The Danish National Prescription Registry
Finland	Statistics Finland	Finland Cause‐of‐Death Register, Statistics Finland	Finnish Hospital Discharge Register	Social Insurance Institute's (KELA) register on reimbursed prescriptions
France: Paris	French National Institute of Statistics and Economic Studies (INSEE)	Vital statistics and mortality records		
Germany: Saxony Anhalt	No	EUROCAT Congenital Anomaly file manually crossed‐checked to mortality records		
Italy: Emilia Romagna	Regional health authority	Regional Mortality Registry (RMR), available from 1995. Regional Inhabitant Registry (RIR), available from 2003	Hospital Discharge Data (SDO), Certificate of Delivery Care (CedAP) which is the main source of public health and statistical data related to birth records.	Assistenza Farmaceutica Territoriale (AFT) translated as Pharmaceutical Territorial Assistance and Farmaci a Erogazione Diretta (FED) translated as Pharmaceutical hospital prescribing
Italy: Tuscany	Regional health authority	Regional Mortality Registry (RMR). Regional Registry Office	Hospital Discharge Records + Birth certificates	Assistenza Farmaceutica Territoriale (AFT) Farmaci a Erogazione Diretta (FED)
Malta	No	Mortality Register		
Netherlands (North)	Dutch Statistics (Central Bureau of Statistics)	Central Bureau of Statistics (CBS)	Dutch Hospital Data. From 2013, the Dutch National Hospital Registration system known as LMR was changed/modernised to LBZ	‘Medicijntab’ available at Dutch Statistics. It includes prescriptions reimbursed by the health insurance companies
Norway	Statistics Norway	Medical Birth Registry of Norway (MBRN), Cause of Death registry		
Spain: Basque Country	No	Spanish mortality database, Registro de Mortalidad		
Spain: Valencian Region	PROSIGA Commission	Regional Mortality Registry and National Mortality Register	Hospital Discharge Records	Integral management of pharmaceutical services module from the Valencian Region (Known as GAIA)
Ukraine	No	Regional Children Hospital Statistics		
UK: East Midlands & South Yorkshire, Thames Valley, Wessex	No	Hospital Episode Statistics (HES) Office of National Statistics (ONS) mortality data	Hospital Episode Statistic (HES), Office of National Statistics (ONS) databases	
UK: Wales	Secure Anonymised Information linkage (SAIL)	Office for National Statistics (ONS)/ National Health System Wales Informatics Service (NWIS)	Patient Episode Database for Wales (PEDW)	Primary care general practitioner (GP) dataset

*Note:* Greyed out columns indicated that a registry did not link to these data sources.

Routinely available healthcare data (birth records, death records, prescription records and hospital discharge records) in each county/region were standardised to common data models developed during the lifetime of the project and central analysis scripts produced aggregate tables for analysis. Individual data on children remained at local registry level. Data were included from 1 January 1995 (or the 1st year with linked data available in each registry), and children were followed up to their 10th birthday or to 31 December 2015 whichever came sooner so that each child had at least 1‐year follow‐up information.

**FIGURE 1:**
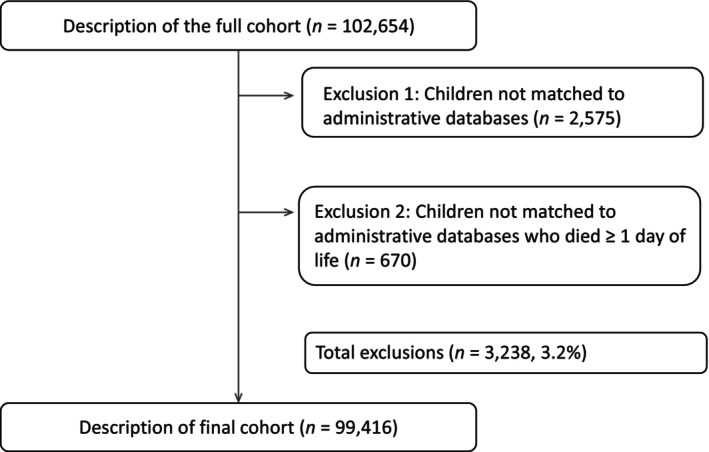
Flow chart describing cohort of children with congenital anomalies.

Registries used either deterministic or probabilistic or a combination of both methods to link the children with anomalies to the healthcare data. Overall, 97% of children from the EUROCAT registries were successfully matched to their records in their national or regional administrative databases (Figure 1), although this varied by region. Detailed information on the methodology used in this study has previously been published [3, 5, 6].

The EUROlinkCAT project was conducted to assess mortality and morbidity outcomes in children with anomalies. To date, 35 peer‐reviewed papers have been published detailing our results which were used to inform these recommendations (Appendix S1).

### 2.1 Ethics approval

Ethics approval for this study was given by the Ulster University Institute of Nursing and Health Research Ethics Filter Committee (FCNUR), approval number: FCNUR‐21‐060.

## 3 Results

### 3.1 Recommendations for Healthcare Databases to Improve the Quality and Use of Data on Anomalies

1. In the mother's record, report ICD codes for terminations of pregnancy for fetal anomalies (TOPFA). Create an additional record, possibly in a separate database, which reports each specific anomaly diagnosed in the fetus and which can be linked to the mother's record.

Surveillance of anomalies is not complete unless terminations of pregnancy following prenatal diagnosis of a fetal anomaly (TOPFA) are included, as approximately 20% of pregnancies with a congenital anomaly in Europe will result in TOPFA [8]. We evaluated the quality and accuracy of codes identifying TOPFA cases in hospital databases in three registries (Finland, Funen [Denmark] and Northern Netherlands) compared with the codes recorded in their EUROCAT databases. Based on data for 2114 TOPFA cases, we found that the percentage of cases for whom there was a code for a congenital anomaly was 90%, 67% and 44% in the three countries. The proportion of TOPFA cases with a code for a specific anomaly was < 50% for cases with a structural anomaly (range: 0%–50%) and 70% for cases with a chromosomal anomaly [2].

These results support our recommendation that it is important that all anomalies leading to the decision to terminate the pregnancy are recorded in the mother's healthcare records. This is challenging as hospital databases often have limited information or codes to identify pregnancies resulting in TOPFA. The World Health Organization (WHO) International Classification of Diseases version 10th revision (ICD‐10) code O04: termination of pregnancy may be recorded in the mother's records without mentioning the specific anomalies. The WHO ICD‐10 has only two potential codes for classifying anomalies that may result in TOPFA in the mother's record: O350 Maternal care for (suspected) central nervous system malformation in fetus and O351 Maternal care for (suspected) chromosomal abnormality in fetus. However, these two codes are also used as a reason for hospitalisation, observation or other obstetric care of the mother, so verification of the TOPFA is needed.

Furthermore, in hospital databases the mother is the patient with the healthcare record, rather than the fetus with the anomalies. This means that results of genetic tests/post‐mortem examinations confirming the congenital anomaly diagnosis are not visible in the hospital databases as the fetus has no healthcare number to record these. This is particularly relevant for surveillance of anomalies with a high termination rate, such as spina bifida, certain heart and renal anomalies and chromosomal anomalies [2]. Precise procedures for reporting all anomalies diagnosed in TOPFAs in hospital databases need to be developed. If the anomalies are recorded in a separate database, then it is crucial that the mother's unique identification (ID) number is also included so that the anomalies diagnosed in the fetus can be linked to the mother.

2 Develop registration systems to assign a permanent unique ID number to each baby as soon as possible after the birth to ensure that all outcomes, procedures and diagnoses occurring during the first days after birth can be linked to the baby.

The availability of a unique ID number present in all routine healthcare databases is essential for successful data linkage studies using deterministic methods, as it enables individuals in one database to be accurately matched to their records in other databases. In addition, the existence of a single data provider who is responsible for all data linkages, is valuable.

We evaluated the rates of linking data on children with anomalies (*n* = 102,654) to regional or national hospital discharge database based on data from 11 registries. We found that 97% of children with anomalies (*n* = 99,416) were successfully matched to their records in their national or regional administrative databases, range: 44%– 99% [5, 6]. Rates varied by region, with registries using deterministic methods having generally the highest rates of successfully linking children to their national vital statistics and healthcare databases. Linkage success was > 99% in Denmark (Funen County) and Finland, 99% in Valencian Region and 95% in the Northern Netherlands. In these countries, everyone in the population is allocated a unique ID number at birth which remains with them until death, and a single data provider links the data.

Emilia Romagna, the three English registries and Wales used a combination of deterministic and probabilistic linkage methods. Their linkage success rates were 93%, 97%, 96%, 91% and 99%, respectively. Despite using both deterministic and probabilistic linkage methods, the two Italian and three English registries were unable to link > 15% of children with anomalies in the earlier years, which meant that data from these years were not included in the EUROlinkCAT studies. Tuscany used probabilistic methods only with a linkage success of 88%. One registry used manual linkage, which is not recommended.

When assessing survival rates, we found that ID numbers were less likely to be allocated to preterm babies who died in the first 48 h after birth in some countries/regions, such as Emilia Romagna (Italy) and Valencian Region (Spain). Some of these early deaths were known in the EUROCAT registries, but were not linked to the official death statistics either because of the linkage methods used or because the death was not registered, or because the baby was transferred to a neonatal intensive care unit outside the region and the death was registered in that jurisdiction. This has important implications for surveillance and research on anomalies due to the potential for bias [5]. For surviving children, we found that 399 children born with oesophageal atresia who were alive 28 days after the birth were identified in the hospital discharge databases. Children with oesophageal atresia need surgery within the first 28 days to survive. However, for 91 (23%) of these children, the code for the neonatal surgery was not visible in the hospital databases. This highlights the need for an ID number to be assigned to each baby as soon as possible after birth so that all outcomes, diagnoses and procedure codes can be linked to the baby's record.

3 Include outpatient contacts in health care databases as less severe congenital anomalies may not be visible in hospital discharge databases if surgery is not required.

We evaluated the accuracy (sensitivity) of coding congenital anomalies in hospital databases by comparing the codes in the hospital databases with the codes in the associated EUROCAT registries, which were assumed to be the gold standard as registries use multiple sources to ascertain cases. Based on data from 45,323 children with anomalies in 11 registries that were linked to hospital records, we found that children with anomalies, such as cleft lip with or without cleft palate, and gastroschisis were accurately identified in hospital inpatient databases (pooled sensitivity > 89%), as these anomalies require surgery. Anomalies not requiring hospitalisation or surgery (e.g., microcephaly, atrial septal defect (ASD), unilateral renal agenesis and hip dislocation) were often under‐reported in hospital in‐patent databases [1]. We also found that Finland and Funen, Denmark, correctly identified children with clubfoot and hydronephrosis (higher sensitivity) compared with the lower sensitivity reported in the other registries, which can be explained by the inclusion of outpatient data in the Finnish and Danish hospital databases.

For completeness of ascertainment of anomalies using healthcare databases, it is important to include data on outpatient appointments if available, as not all children with anomalies require inpatient stays or surgery; many will be seen at outpatient clinics to monitor progress and development. Also, if children have surgery outside their region (e.g., in a specialised centre), they may be followed up at an outpatient clinic in their region enabling the anomalies to be accurately reported.

A complication relevant to twin pregnancies is that a prenatal diagnosis of a severe anomaly in one fetus may be followed by a fetal reduction procedure which is usually performed in the outpatient clinic. Therefore, the subsequent birth of the co‐twin may be recorded as a singleton birth. The WHO ICD‐10 code O31.1 or the ICD‐10‐CM code O31.30 can be used to code the woman continuing pregnancy after elective fetal reduction in one fetus or more. By including outpatient data, it is possible to identify these women if the code for the fetal reduction is recorded and linkage to obtain additional data on the anomalies diagnosed in the fetus can occur.

4 Allow the use of more than five diagnosis codes for both hospital discharges and outpatient contacts.

This recommendation is a software issue which is particularly relevant to surveillance of multiple anomalies as around 25% of children have more than one anomaly [9]. There are many clinical situations that require > 5 diagnoses at discharge from a neonatal intensive care unit, and the hospital database must have the functionality to allow all the diagnoses to be entered. For example: a baby with Down syndrome and atrioventricular septal defect (AVSD) born preterm with low birth weight has respiratory problems and treatment for sepsis. Recorded diagnoses would be P073 preterm, P071 low birth weight, P220 respiratory distress syndrome, P369 neonatal sepsis, P590 jaundice, Q900 Down syndrome, and Q2120 AVSD.

If a hospital database only allows one discharge code, ascertainment of anomalies will be incomplete using these databases and cases will be more likely to be classified as isolated due to the inability to record co‐occurring defects [10]. Furthermore, if there is a limit on the number of recorded diagnosis codes in a hospital database, a decision is needed by the doctor or coder as to which is the correct diagnosis to be recorded in the database. For instance, a code for preterm birth or for the infection that was the leading cause for the hospital stay may be recorded rather than the congenital anomaly [11].

5 Allow codes to be revised within a certain time frame rather than adding an updated diagnosis, as the coding may be amended by more experienced doctors and coders; or the coding may be refined by results of diagnostic examinations/tests arriving after the TOPFA or after the child has left the hospital.

To improve the accuracy of congenital anomaly coding in healthcare databases, it is important to allow diagnoses to be amended when updated information is available. For example, codes recorded at birth or at initial hospital visits may reflect a suspected or differential diagnosis, for example, a diagnosis of hip dislocation or hypospadias may be suspected by the midwife at birth and coded at discharge, but it may not be confirmed or rejected until the baby is referred to paediatricians or surgical departments for evaluation. Diagnoses may also be updated following receipt of test results confirming the final diagnosis, such as genetic tests for karyotype anomalies, genetic tests for rare syndromes or a biopsy to confirm the diagnosis of Hirschsprung. Post‐mortem examination after a TOPFA or neonatal death may also show additional major anomalies.

EUROlinkCAT partners were asked to send a short survey on coding practices to hospital doctors in their registry. A total of 73 questionnaires were received from 11 registry areas. The survey found that in some countries, such as Denmark, the medical doctors code all diagnoses for the discharge letter, whereas in others, such as the United Kingdom (UK), trained coders read the medical record after discharge and add the relevant codes for each hospital stay (Table 2). An important finding was that responses from Germany, Italy, Netherlands and Spain stated that no ICD codes were given for an outpatient visit.

**TABLE 2:** Results of coding practice in hospital discharge databases in EUROlinkCAT regions.

EUROCAT Registry	Responses (*n*)	Coding by trained coders (*n*)	Coding by doctors (*n*)	ICD codes recorded in outpatient visits
Belgium—Antwerp	2	2	0	Yes
Croatia—Zagreb	1	0	1	Yes
Denmark—Funen	4	0	4	Yes
Finland	1	0	1	Yes
Germany—Saxony Anhalt	7	7	4	No
Italy—Tuscany	4	0	4	No
Northern Netherlands	1	1	0	No
Poland	45	2	43	Yes
South Portugal	5	5	5	Yes
Spain—Valencian Region	2	2	0	No
UK	1	1	0	Yes
Total	73	20	62	

Different doctors involved in the treatment and follow‐up of a child with an anomaly, both within and between hospitals, may use different codes to code the same anomaly or may use unspecified codes. This is particularly challenging if there is no international consensus on a definition of a diagnosis, for example, severe congenital heart defects [12]. Medical doctors participating in a focus group study exploring factors affecting the quality of coded data in health records reported variability in diagnosis description by different health professionals as a potential data quality issue [13].

**FIGURE 2:**
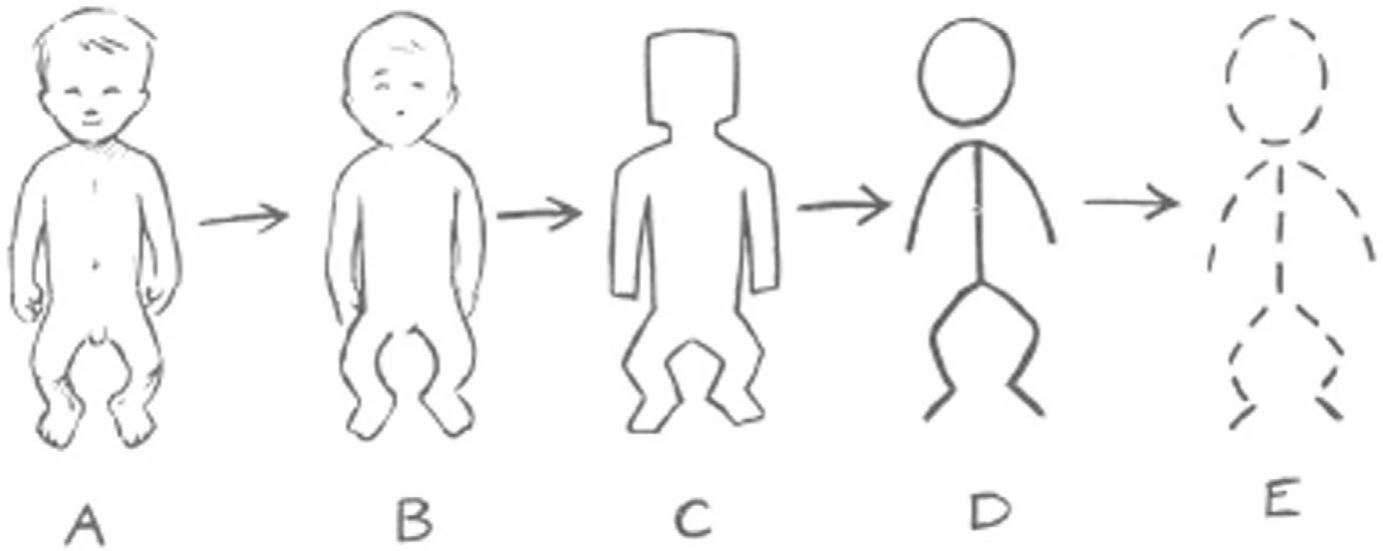
Distortion of information in a reporting system—from presentation of infant to coded data. (A) shows the actual infant, (B) is the doctor's picture of it and what is written down in the medical records, (C) is the content of the report form to the surveillance registry, (D) is the interpretation of that form in the registry, and (E) is the coded data which are stored in a computer [14].

The quality of the coding of diseases in health care databases is dependent on factors, such as the quality of the coding system used and how detailed it is, the clinical knowledge of the coder, the time available for coding and the diagnostic details available about the patient. Some level of diagnostic detail will be lost when using a code compared with the detailed text description available in the medical notes (Figure 2). Nonetheless, avoiding the use of ‘unspecified’ or ‘other specified’ codes in hospital databases will improve the coding of anomalies.

6 Use extended versions of ICD for the coding of rare congenital anomalies or use other coding systems to make the rare diagnoses visible in health care databases.

The WHO ICD‐10 Q‐chapter has been used for coding of anomalies in EUROCAT since 2005 and greater specificity of anomaly coding is obtained by using the British Pediatric Association (BPA) extension codes [3]. These extension codes are not available in hospital databases so surveillance using health care databases would be unable to identify children with, for example, Q2110 ASD secundum and Q2111 persistent foramen ovale or associations, such as VATER (Q8726) and sequences (Pierre Robin Q8708 and sirenomelia Q8724). The new ICD‐11, in use from 2022, has a chapter on extension codes which enables more detailed information to be added to the ‘stem’ code [15]. However, the efficacy of coding rare anomalies in ICD‐11 still has to be tested.

## 4 Recommendations for Registries to Improve the Quality and Use of Data on Anomalies

7 Incorporate validated coding algorithms to identify congenital anomalies within the databases.

We also evaluated the specificity of coding congenital anomalies in healthcare databases. Based on data from five registries (*n* = 24,066 children linked to hospital records), we found that children with anomalies, such as hypoplastic left heart (HLH), spina bifida, Hirschsprung's disease, omphalocele and cleft palate were over‐reported in the hospital data which suggests that the hospital data contained some false positives (1). For instance, the overall pooled positive predictive value (PPV) estimate was 71% for HLH which means that 29% of children with HLH codes in the hospital data did not have this heart anomaly according to EUROCAT (false positive hospital cases).

These results corroborate the findings in Recommendation 3 that the coding of anomalies is not sufficiently accurate in administrative databases; therefore, the use of algorithms to discriminate between true anomalies and suspected or minor cases is recommended. For example, an algorithm that requires surgery to be performed for anomalies, such as craniosynostosis, choanal atresia, Hirschsprung's anomaly or hypospadias to be defined as a case would improve diagnostic accuracy. The Emilia Romagna registry in Italy developed an algorithm for ascertaining anomalies in newborn children registered in their regional healthcare databases. This algorithm was effective in reducing the number of cases to be manually evaluated, without greatly increasing the probability of error in the validated cases (false positives) and in those excluded (false negatives) [16]. Although healthcare databases can be used to identify children with anomalies, they should not be used as the only source due to inaccurate coding.

8 Determine the precise small number suppression requirements from each database to ensure meaningful results will be obtainable from that database.

Many databases had strict rules regarding publishing tables or analytical results based on small numbers of cases, due to potential risks of disclosure. For example, cells with < 3 cases could not be released for Belgium (Antwerp) and Denmark, < 5 for Wales and < 8 for England. In the Northern Netherlands, all counts had to be rounded to the nearest multiple of 5.

Preventing the release of results based on a small number of children was severely deleterious to the analysis of data on rare anomalies as, by definition, many registries would only have a limited number of cases; hence, the need to combine data across registries. We advocate that national statistics organisations should release small numbers to named trusted researchers on the study team who have signed additional disclosure agreements, on the condition that these results will only be published when combined with results from other countries/regions.

## 5 Comment

EUROlinkCAT highlighted the many challenges and opportunities inherent in analysing healthcare data across several countries/regions in Europe. Data routinely collected in electronic healthcare databases should be improved to enable the data to be used in the surveillance and research of anomalies. Codes for classifying and reporting anomalies resulting in TOPFAs in healthcare databases need to be developed. In addition, the accuracy of the coding of anomalies in all births should be improved and algorithms to accurately discriminate between major congenital anomalies and suspected or minor anomalies should continue to be refined.

## 6 Conclusion

EUROlinkCAT demonstrated that healthcare databases contain valuable data on mortality and morbidity outcomes in children with anomalies, but that they currently cannot be used as the only data source for the surveillance of anomalies.

## Author Contributions

Maria Loane, Joan K Morris and Ester Garne conceptualised the EUROlinkCAT study and obtained funding. Maria Loane and Joan Morris co‐wrote the first draft of the manuscript. All authors commented on previous versions of the manuscript and read and approved the final manuscript.

## Ethics Statement

A study protocol was developed for EUROCAT registries to obtain local ethics and governance approval for the study according to their national legislation.^1^ Ethics approval for this study was given by the Ulster University Institute of Nursing and Health Research Ethics Filter Committee (FCNUR), approval number: FCNUR‐21‐060.

## Consent

As each registry uploaded aggregate data only to the research team, individual consent was not required, as no individual could be identified from the uploaded tables.

## Conflicts of Interest

The authors declare no conflicts of interest.

## Supporting information


Appendix S1.

## Data Availability

The data that support the findings of this study are available, but restrictions apply to the availability of these data, which were used under licence for the current study and so are not publicly available. Data are, however, available from the authors after the permission of the participating registries of congenital anomalies.
