# Supplementary material for: Recommendations for Improving Surveillance of Congenital Anomalies in Europe Using Healthcare Databases
Source: Paediatr Perinat Epidemiol. 2025 Jan 28;39(3):277–84. doi: 10.1111/ppe.13173 (PMC11997239; doi:10.1111/ppe.13173)
Supplement: Supplementary file 1 — Appendix S1. [file PPE-39-277-s001.docx]

**Supplementary Appendix 1: List of EUROlinkCAT publications**

1. Urhoj SK, Morris J, Loane M, et al. Higher risk of cerebral palsy, seizures/epilepsy, visual- and hearing impairments, cancer, injury and child abuse in children with congenital anomalies: Data from the EUROlinkCAT study. *Acta Paediatrica* 2024;n/a(n/a) doi: [https://doi.org/10.1111/apa.17136](https://protect.checkpoint.com/v2/___https://doi.org/10.1111/apa.17136___.YzJlOnVsc3RlcnVuaXZlcnNpdHk6YzpvOmQ0MTg0OGQ0OTczZmZjNzhkOTIzMTAzOGIwM2U5YWQyOjY6MWUwODpiOGYxNmVmYzEyMzZkYjNjNmRkZjVmZTg5YWJkNmRlYWM4ZTE3YjJkN2ZhYzc5ZmQ5MWMxNGEyMWRkZDY5NDUxOnA6VDpO)

2. Morris JK, Loane M, Wahlich C, et al. Hospital care in the first 10 years of life of children with congenital anomalies in six European countries: data from the EUROlinkCAT cohort linkage study. *Arch Dis Child* 2024;109(5):402-08. doi: 10.1136/archdischild-2023-326557 [published Online First: 20240418]

3. Heino A, Morris JK, Garne E, et al. The Association of Prenatal Diagnoses with Mortality and Long-Term Morbidity in Children with Specific Isolated Congenital Anomalies: A European Register-Based Cohort Study. *Maternal and Child Health Journal* 2024;28(6):1020-30. doi: 10.1007/s10995-024-03911-9

4. Glinianaia SV, Tan J, Morris JK, et al. Academic achievement at ages 11 and 16 in children born with congenital anomalies in England: A multi-registry linked cohort study. *Paediatric and Perinatal Epidemiology* 2024;n/a(n/a) doi: [https://doi.org/10.1111/ppe.13049](https://protect.checkpoint.com/v2/___https://doi.org/10.1111/ppe.13049___.YzJlOnVsc3RlcnVuaXZlcnNpdHk6YzpvOmQ0MTg0OGQ0OTczZmZjNzhkOTIzMTAzOGIwM2U5YWQyOjY6Y2Q0NjozMGI3YjBiNDIxN2NiNzY3YzNmYTBjMjY0MzI5ZmU3Y2Y3ZGMzNWUxYTIzMjg5OGIzODk0YWYzM2Y3NjI5YTA4OnA6VDpO)

5. Damkjær M, Tan J, Morris JK, et al. Children with Hirschsprung's disease have high morbidity in the first 5 years of life. *Birth Defects Research* 2024;116(5):e2338. doi: [https://doi.org/10.1002/bdr2.2338](https://protect.checkpoint.com/v2/___https://doi.org/10.1002/bdr2.2338___.YzJlOnVsc3RlcnVuaXZlcnNpdHk6YzpvOmQ0MTg0OGQ0OTczZmZjNzhkOTIzMTAzOGIwM2U5YWQyOjY6ZjNmOTplN2E3MWQ0OWMxMWM3NGU5NWViY2I1OWFjMzIyZmQ3Nzk4MDUwYzVkZjJjNDgwMDFhMTg4YWVkMTg0YmViNmM3OnA6VDpO)

6. Tan J, Glinianaia SV, Rankin J, et al. Risk factors for mortality in infancy and childhood in children with major congenital anomalies: A European population-based cohort study. *Paediatr Perinat Epidemiol* 2023 doi: 10.1111/ppe.13010 [published Online First: 20231010]

7. Santoro M, Garne E, Coi A, et al. Survival, hospitalisation and surgery in children born with Pierre Robin sequence: a European population-based cohort study. *Arch Dis Child* 2023;108(7):550-55. doi: 10.1136/archdischild-2022-324716 [published Online First: 20230509]

8. Roustaei Z, Heino A, Kiuru-Kuhlefelt S, et al. Educational achievement of children with selected major congenital anomalies and associated factors: a Finnish registry-based study. *Eur J Public Health* 2023 doi: 10.1093/eurpub/ckad149 [published Online First: 20230818]

9. Rissmann A, Tan J, Glinianaia SV, et al. Causes of death in children with congenital anomalies up to age 10 in eight European countries. *BMJ Paediatr Open* 2023;7(1) doi: 10.1136/bmjpo-2022-001617

10. Loane M, Given JE, Tan J, et al. Creating a population-based cohort of children born with and without congenital anomalies using birth data matched to hospital discharge databases in 11 European regions: Assessment of linkage success and data quality. *PLOS ONE* 2023;18(8):e0290711. doi: 10.1371/journal.pone.0290711

11. Glinianaia SV, Rankin J, Tan J, et al. Ten-year survival of children with trisomy 13 or trisomy 18: a multi-registry European cohort study. *Arch Dis Child* 2023 doi: 10.1136/archdischild-2022-325068 [published Online First: 2023/03/08]

12. Given J, Morris JK, Garne E, et al. Prescriptions for insulin and insulin analogues in children with and without major congenital anomalies: a data linkage cohort study across six European regions. *Eur J Pediatr* 2023 doi: 10.1007/s00431-023-04885-6 [published Online First: 2023/03/04]

13. Garne E, Urhoj SK, Bakker M, et al. The quality and the accuracy of codes for terminations of pregnancy for fetal anomalies recorded in hospital databases in three countries in northern Europe. *Birth Defects Res* 2023;115(3):405-12. doi: 10.1002/bdr2.2133 [published Online First: 2022/12/28]

14. Garne E, Tan J, Damkjaer M, et al. Hospital Length of Stay and Surgery among European Children with Rare Structural Congenital Anomalies-A Population-Based Data Linkage Study. *Int J Environ Res Public Health* 2023;20(5) doi: 10.3390/ijerph20054387 [published Online First: 2023/03/12]

15. Garne E, Loane M, Tan J, et al. European study showed that children with congenital anomalies often underwent multiple surgical procedures at different ages across Europe. *Acta Paediatrica* 2023;n/a(n/a) doi: [https://doi.org/10.1111/apa.16726](https://protect.checkpoint.com/v2/___https://doi.org/10.1111/apa.16726___.YzJlOnVsc3RlcnVuaXZlcnNpdHk6YzpvOmQ0MTg0OGQ0OTczZmZjNzhkOTIzMTAzOGIwM2U5YWQyOjY6ODFhMjo4N2M3ZThlZWUzODcwOWQ3YTc4OTUzNDMyMGNiZjlhYjk4NDNmMzc0NmM2NzY5ZGExN2FmNjUyODgzMzUzYzg2OnA6VDpO)

16. Divin N, Given JE, Tan J, et al. Antiasthmatic prescriptions in children with and without congenital anomalies: a population-based study. *BMJ Open* 2023;13(10):e068885. doi: 10.1136/bmjopen-2022-068885

17. Damkjær M, Garne E, Loane M, et al. Timing of Cardiac Surgical Interventions and Postoperative Mortality in Children With Severe Congenital Heart Defects Across Europe: Data From the EUROlinkCAT Study. *J Am Heart Assoc* 2023:e029871. doi: 10.1161/jaha.122.029871 [published Online First: 20231218]

18. Claridge H, Tan J, Loane M, et al. Ethics and legal requirements for data linkage in 14 European countries for children with congenital anomalies. *BMJ Open* 2023;13(7):e071687. doi: 10.1136/bmjopen-2023-071687

19. Bakker MK, Loane M, Garne E, et al. Accuracy of congenital anomaly coding in live birth children recorded in European health care databases, a EUROlinkCAT study. *Eur J Epidemiol* 2023 doi: 10.1007/s10654-023-00971-z [published Online First: 2023/02/23]

20. Andersen A-LR, Urhoj SK, Tan J, et al. The burden of disease for children born alive with Turner syndrome—A European cohort study. *Birth Defects Research* 2023;115(16):1459-68. doi: [https://doi.org/10.1002/bdr2.2222](https://protect.checkpoint.com/v2/___https://doi.org/10.1002/bdr2.2222___.YzJlOnVsc3RlcnVuaXZlcnNpdHk6YzpvOmQ0MTg0OGQ0OTczZmZjNzhkOTIzMTAzOGIwM2U5YWQyOjY6ZGJhYzplN2VjNDEwYTBhNzNjYTdhM2NkM2Q1NTY4YzE4ZmEwMmU5ZGZjOTE5NTQ4ZDJhN2UwODFjYjY0ZjNhMjhmODFjOnA6VDpO)

21. Urhoj SK, Tan J, Morris JK, et al. Hospital length of stay among children with and without congenital anomalies across 11 European regions—A population-based data linkage study. *PLoS One* 2022;17(7):e0269874. doi: 10.1371/journal.pone.0269874

22. Santoro M, Coi A, Pierini A, et al. Temporal and geographical variations in survival of children born with congenital anomalies in Europe: A multi-registry cohort study. *Paediatric and Perinatal Epidemiology* 2022;36(6):792-803. doi: [https://doi.org/10.1111/ppe.12884](https://protect.checkpoint.com/v2/___https://doi.org/10.1111/ppe.12884___.YzJlOnVsc3RlcnVuaXZlcnNpdHk6YzpvOmQ0MTg0OGQ0OTczZmZjNzhkOTIzMTAzOGIwM2U5YWQyOjY6YjEwMDo2NDhlYjg3ZGYyNWEyYjU5ZWIzZjk2MjE0ZWVlNDVjOGZkYWY3OTA2NzUxM2NkMzRlNzE0ZWVhMzg1OTI3YzhmOnA6VDpO) [published Online First: 20220608]

23. Marcus E, Latos-Bielenska A, Jamry-Dziurla A, et al. Information needs of parents of children with congenital anomalies across Europe: a EUROlinkCAT survey. *BMC Pediatrics* 2022;22(1):657. doi: [https://dx.doi.org/10.1186/s12887-022-03734-z](https://protect.checkpoint.com/v2/___https://dx.doi.org/10.1186/s12887-022-03734-z___.YzJlOnVsc3RlcnVuaXZlcnNpdHk6YzpvOmQ0MTg0OGQ0OTczZmZjNzhkOTIzMTAzOGIwM2U5YWQyOjY6NGQyZDpkODVmODdmOWE4YmY3ZTNhNjA4ZjkzOWRlNTQwYThhYmUyNWYzMTQ0MjE1OWI5MjNkMDBlOTY4NDAwN2E0OGYzOnA6VDpO)

24. Latos-Bielenska A, Marcus E, Jamry-Dziurla A, et al. COVID-19 and children with congenital anomalies: a European survey of parents' experiences of healthcare services. *BMJ Open* 2022;12(7):e061428. doi: [https://dx.doi.org/10.1136/bmjopen-2022-061428](https://protect.checkpoint.com/v2/___https://dx.doi.org/10.1136/bmjopen-2022-061428___.YzJlOnVsc3RlcnVuaXZlcnNpdHk6YzpvOmQ0MTg0OGQ0OTczZmZjNzhkOTIzMTAzOGIwM2U5YWQyOjY6YTMyMjo4NzlhYThhMzRjZjEyZTcyY2M2MjU0OTFmZmRmYzUwMjMxODA4ZTI0YjAwY2NmMzQ5OTQ5Y2ZmYTY1ODBmOWMzOnA6VDpO)

25. Glinianaia SV, Rankin J, Pierini A, et al. Ten-Year Survival of Children With Congenital Anomalies: A European Cohort Study. *Pediatrics* 2022;149(3) doi: 10.1542/peds.2021-053793 [published Online First: 2022/02/12]

26. Garne E, Tan J, Loane M, et al. Gastrostomy and congenital anomalies: a European population-based study. *BMJ Paediatrics Open* 2022;6(1):e001526. doi: 10.1136/bmjpo-2022-001526

27. Damkjaer M, Urhoj SK, Tan J, et al. Prescription of cardiovascular medication in children with congenital heart defects across six European Regions from 2000 to 2014: data from the EUROlinkCAT population-based cohort study. *BMJ Open* 2022;12(4):e057400. doi: 10.1136/bmjopen-2021-057400 [published Online First: 2022/04/23]

28. Damkjaer M, Loane M, Urhoj SK, et al. Preterm birth and prescriptions for cardiovascular, antiseizure, antibiotics and antiasthmatic medication in children up to 10 years of age: a population-based data linkage cohort study across six European regions. *BMJ Open* 2022;12(10):e061746. doi: [https://dx.doi.org/10.1136/bmjopen-2022-061746](https://protect.checkpoint.com/v2/___https://dx.doi.org/10.1136/bmjopen-2022-061746___.YzJlOnVsc3RlcnVuaXZlcnNpdHk6YzpvOmQ0MTg0OGQ0OTczZmZjNzhkOTIzMTAzOGIwM2U5YWQyOjY6ZDViMDoxOGFmNTExNmNlMTZiYzY4ZmM5ODNhM2YyNzdkYWVhZjQxYjE3YzVmYTQ0NzEwMWFmOWNiYWYzNWJkOTRlYjMyOnA6VDpO)

29. Coi A, Santoro M, Pierini A, et al. Survival of children with rare structural congenital anomalies: a multi-registry cohort study. *Orphanet Journal of Rare Diseases* 2022;17(1):142. doi: 10.1186/s13023-022-02292-y

30. Morris JK, Garne E, Loane M, et al. EUROlinkCAT protocol for a European population-based data linkage study investigating the survival, morbidity and education of children with congenital anomalies. *BMJ Open* 2021;11(6):e047859. doi: 10.1136/bmjopen-2020-047859

31. Loane M, Given JE, Tan J, et al. Linking a European cohort of children born with congenital anomalies to vital statistics and mortality records: A EUROlinkCAT study. *PLoS One* 2021;16(8):e0256535. doi: 10.1371/journal.pone.0256535

32. Holm KG, Neville AJ, Pierini A, et al. The Voice of Parents of Children With a Congenital Anomaly - A EUROlinkCAT Study. *Front Pediatr* 2021;9:654883. doi: 10.3389/fped.2021.654883 [published Online First: 2021/12/17]

33. Glinianaia SV, McLean A, Moffat M, et al. Academic achievement and needs of school-aged children born with selected congenital anomalies: A systematic review and meta-analysis. *Birth Defects Res* 2021;113(20):1431-62. doi: 10.1002/bdr2.1961 [published Online First: 20211021]

34. Glinianaia SV, Morris JK, Best KE, et al. Long-term survival of children born with congenital anomalies: A systematic review and meta-analysis of population-based studies. *PLOS Medicine* 2020;17(9):e1003356. doi: 10.1371/journal.pmed.1003356

35. Sinclair M, McCullough JE, Elliott D, et al. Exploring Research Priorities of Parents Who Have Children With Down Syndrome, Cleft Lip With or Without Cleft Palate, Congenital Heart Defects, or Spina Bifida Using ConnectEpeople: A Social Media Coproduction Research Study. *J Med Internet Res* 2019;21(11):e15847. doi: 10.2196/15847 [published Online First: 2019/11/26]
